# Testicular Rupture: A Tough Nut to Crack

**DOI:** 10.5811/cpcem.2017.3.33348

**Published:** 2017-07-06

**Authors:** Tyler L. Holliday, Kristine S. Robinson, Nicole Dorinzi, Andrew W. Vucelik, Erin L. Setzer, Debra L. Williams, Melinda J. Sharon, Joseph J. Minardi

**Affiliations:** * West Virginia University, School of Medicine, Morgantown, West Virginia; † West Virginia University, Department of Emergency Medicine, Morgantown, West Virginia

## Abstract

Blunt scrotal injury represents a diagnostic dilemma for emergency physicians (EP). Consequently, point-of-care ultrasound (POCUS) has emerged as a tool for early investigation of the acute scrotum in the emergency department. We describe a case where an EP used scrotal POCUS to immediately visualize the loss of testicular contour and underlying heterogeneous parenchyma to rapidly make the diagnosis of testicular rupture in a young male presenting with scrotal trauma. The use of POCUS in this case expedited therapy, likely improving the patient’s outcome. To our knowledge, this is the first detailed description of testicular rupture diagnosed with POCUS by an EP

## INTRODUCTION

Acute scrotal pain is a common complaint in the emergency department (ED).^1^,^2^ Etiologies of the acute scrotum include testicular torsion, infection, and trauma. The majority of traumatic injuries are blunt impacts, most commonly in adolescents and young adults.^2^–^4^ Evaluation of these patients poses a significant challenge, as history and physical examination findings are often equivocal. Thus, point-of-care ultrasound (POCUS) has increasingly been used for early investigation of the acute scrotum.^1^ Many studies have shown scrotal US to be fast and reliable in differentiating a broad range of time-sensitive pathologies, including testicular torsion, fracture, and rupture, as well as differentiating surgical and non-surgical emergencies where delays in care may lead to poor outcomes.^1^,^3^,^4^–^7^ However, the use of POCUS in these patients by emergency physicians (EP) is limited to a single case series from 2001.^1^ We present a case of testicular rupture diagnosed by an EP using scrotal POCUS that led to expedited care and surgical intervention.

## CASE REPORT

An 18-year-old male presented to the ED with left testicular pain and swelling following blunt scrotal trauma sustained during a wrestling match. The pain was immediate, but did not necessitate removal from play. Post-injury, the pain progressed, prompting presentation to our ED. The patient denied dysuria, hematuria, or any other symptoms. Past medical and surgical histories were unremarkable. Vital signs were normal. On physical examination, the left testis was tender to palpation, grossly swollen, and tense compared to the right. The remaining physical examination, including abdominal exam, was unremarkable.

**Image 1:**
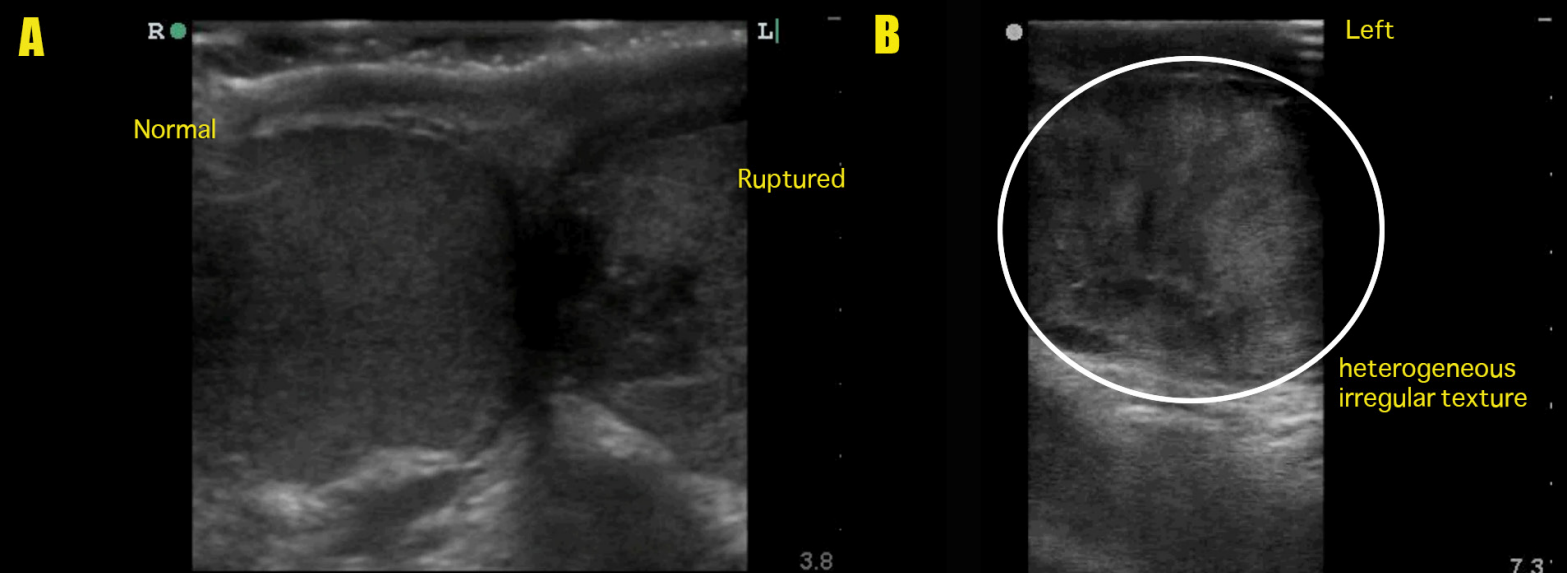
A: Side-by-side comparison of right and left testicles demonstrating normal right testis with homogenous parenchyma, intact tunica albuginea, and preserved testicular contour and ruptured left testis with heterogeneous parenchyma. B: Ruptured left testis. Long-axis view demonstrating heterogeneous irregular parenchyma (circle).

**Image 2:**
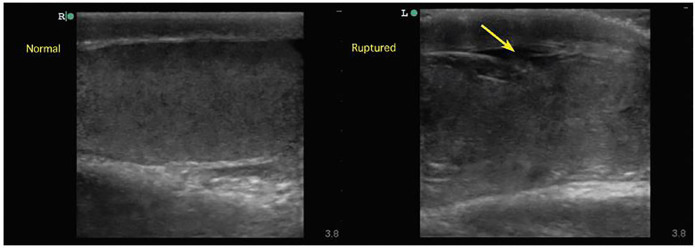
Long-axis comparison of normal and ruptured testicles. Right image demonstrates a normal testis with homogeneous parenchyma, intact tunica albuginea, and preserved testicular contour. Left image demonstrates testicular rupture with findings of heterogeneous parenchyma, discontinuity of tunica albuginea (arrow), and abnormal testicular contour.

Scrotal POCUS was carried out by the EP using a Sonosite Edge system using an L13–6 linear transducer operating at 10 MHz. Sagittal and transverse images of the affected testis along with comparison views of the unaffected side were obtained. Analysis with color and spectral Doppler was performed. The left testis was enlarged with a grossly heterogeneous texture as seen in Image 1 and the Supplemental Video. Discontinuity of the tunica albuginea and irregularity of the testicular margins were noted as seen in Image 2 and the Supplemental Video. Additionally, a hypoechoic complex fluid collection consistent with hematocele was present in the left hemiscrotum, and blood flow was not uniformly identified throughout the left testis. The left epididymis was unremarkable. The right testis appeared normal with homogenous parenchyma, intact tunica albuginea, smooth, preserved borders, and uniform distribution of blood flow throughout by color Doppler analysis (seen in Image 1, panel A, Supplemental Video). No fluid collection was observed within the right hemiscrotum and the right epididymis was unremarkable. A rapid diagnosis of left testicular rupture was made in the ED, and urology was consulted.

The patient was taken to the operating room for scrotal exploration. The left testis was found to be fractured, without viability of the lower segment. All nonviable tissue was removed, and bleeding was controlled. The upper pole was preserved, the tunica albuginea and vaginalis were re-approximated, and the scrotum was closed. The patient was admitted overnight for observation and discharged the following morning. At six-week follow-up, the patient had returned to normal activity.

CPC-EM Capsule

What do we already know about this clinical entity?

Injuries from blunt scrotal trauma are difficult to diagnose. Ultrasound is the preferred imaging modality to visualize scrotal pathologies and differentiate surgical and non-surgical diagnoses.

What makes this presentation of disease reportable?

To our knowledge, this is the first detailed description of testicular rupture diagnosed with point-of-care ultrasound (POCUS) by an emergency physician (EP).

What is the major learning point?

The findings of testicular rupture, including loss of tunica albuginea integrity and testicular heterogeneity can be identified sonographically by EPs in a rapid fashion. Rapid recognition of these findings can influence timing of consultation and expedite surgery, which should improve outcomes.

How might this improve emergency medicine practice?

Rapidly recognizing and/or excluding the findings of testicular rupture can decrease the need for consultative imaging, expedite definitive management and streamline patient care.

## DISCUSSION

Blunt trauma to the scrotum may result in a number of injuries, including hematocele, testicular fracture, and testicular rupture. Typically, these patients will present with non-specific symptoms, such as scrotal swelling and severe pain, which often make physical examination difficult to perform. Historically, blunt scrotal traumas were managed non-operatively, with surgical exploration reserved for cases with resulting complications. Higher rates of adverse outcomes (impaired fertility, hypogonadism, testicular loss) were associated with this delay in care.^8^ In the case of testicular rupture, salvage rates are as high as 90% when surgery is performed within 72 hours. When surgical intervention is delayed beyond 72 hours, salvage rates decline to approximately 45%.^9^

US has emerged as the preferred modality for examining the acute scrotum. It can be rapidly employed to provide high quality imaging that can accurately guide clinical decisions and differentiate surgical from non-surgical pathologies. Scrotal POCUS, in the hands of an EP, provides an opportunity to reduce time to diagnosis and reduce dependence on sonographers and radiology departments, which may not be available at all hours. EPs with significant US experience, even in the absence of formal testicular US training, are highly accurate in diagnosing scrotal pathologies.^1^

Ultrasonic imaging of the scrotum should be undertaken with a high-frequency (7–12MHz) linear transducer. The scrotum should be supported by a towel placed between adducted thighs with the penis displaced away from the scrotum. The hand holding the transducer should be supported against the thigh and a generous amount of gel should be used in order to provide adequate acoustic contact between the scrotum and transducer. Each testis should be visualized in multiple planes of the long and short axes with and without color Doppler. If one structure is abnormal, the contralateral side should be used to calibrate the grayscale and color Doppler gain settings for examination of the symptomatic testicle.^9^

Using ultrasonography, a normal post-pubertal testis should appear as a homogeneous ovoid structure of medium echogenicity measuring approximately 5×3×2cm. The tunica albuginea surrounds the testis and appears as a thin echogenic band. The epididymis should appear as a long, tapering tubular structure bordering the posterior aspect of the testis. Typically, the epididymis is isoechoic to hyperechoic and homogeneous.^4^,^9^

With scrotal POCUS, hematocele, testicular fracture, and testicular rupture can be quickly identified and treated in patients reporting to the ED with blunt scrotal trauma. Hematoceles appear as extratesticular fluid collections of increased echogenicity acutely after traumas but become hypoechoic and septated over time.^4^,^9^ Surgical management is recommended for large (>5 cm) or expanding hematoceles. Otherwise, conservative management is recommended.^10^ Testicular fracture is identified by a linear hypoechoic band that divides the testicular parenchyma within an intact tunica albuginea. If blood flow is preserved, the fractures are treated conservatively (scrotal support, NSAIDs, ice packs, bed rest, and serial ultrasounds). If the segment is avascular, emergent surgical intervention is necessitated.^3^,^4^,^10^ Testicular rupture is identified by loss of testicular contour with underlying heterogeneity of the parenchyma. Diagnosis requires emergent surgery to preserve testicular function.^2^,^4^,^6^,^7^

The accuracy of US for the evaluation of these pathologies has been established through retrospective studies that have compared sonographic findings against those found on subsequent scrotal exploration, the traditional gold standard for diagnostic and therapeutic evaluation.^6^,^8^ The post-traumatic ultrasonic evaluation for hematocele has been shown to be 85–87% sensitive and 75–89% specific according to two studies of 24 and 33 patients, respectively.^6^,^8^ Three studies, consisting of 33, 65, and 24 patients, have reported the sonographic findings for testicular rupture to be 92–100% sensitive while being 50–93.5% specific.^6^–^8^ The accuracy of US in the diagnosis of testicular fracture is less well established since the diagnosis is uncommon and most of these patients are managed non-operatively.

## CONCLUSION

In summary, we report a case of testicular rupture diagnosed by an EP using POCUS. To our knowledge, this diagnosis identified with POCUS has not been detailed in the emergency medicine literature. Our case demonstrates an opportunity for EPs to expedite the diagnosis of acute scrotal pathologies, and reduce dependence on radiology departments, which may not be readily available. Furthermore, use of routine scrotal POCUS may be used to stratify patients in need of emergent urologic consultation, help to reduce time to surgical intervention, and reduce the need for additional evaluation, thus improving resource use and patient outcomes.

## Supplementary Information

Supplemental Video This brief, narrated video reviews the findings in this case which led to the immediate diagnosis of testicular rupture. The findings include enlarged testicle with irregular, heterogeneous echogenicity, as well as loss of integrity of the tunica albuginea.
